# Through the lens of bioenergy crops: advances, bottlenecks, and promises of plant engineering

**DOI:** 10.1111/tpj.70294

**Published:** 2025-07-17

**Authors:** Angel Indibi, Pengfei Cao, Federica Brandizzi, Jenny Mortimer, Kankshita Swaminathan, Chung‐Jui Tsai, Bjoern Hamberger

**Affiliations:** ^1^ Great Lakes Bioenergy Research Center Michigan State University East Lansing Michigan 48824 USA; ^2^ Department of Biochemistry and Molecular Biology Michigan State University East Lansing Michigan USA; ^3^ MSU‐DOE Plant Research Lab Michigan State University East Lansing Michigan 48824 USA; ^4^ Department of Plant Biology Michigan State University East Lansing Michigan 48824 USA; ^5^ Joint BioEnergy Institute Emeryville California 94608 USA; ^6^ Environmental Genomics and Systems Biology Division Lawrence Berkeley National Laboratory One Cyclotron Road, MS 978R4468 Berkeley California 94720 USA; ^7^ School of Agriculture, Food, and Wine University of Adelaide Glen Osmond South Australia Australia; ^8^ HudsonAlpha Institute for Biotechnology 601 Genome Way Huntsville Alabama 35806 USA; ^9^ Center for Advanced Bioenergy and Bioproducts Innovation 1206 W. Gregory Drive (IGB) Urbana Illinois 61801 USA; ^10^ Warnell School of Forestry and Natural Resources University of Georgia Athens Georgia 30602 USA; ^11^ Department of Genetics University of Georgia Athens Georgia 30602 USA; ^12^ Department of Plant Biology University of Georgia Athens Georgia 30602 USA

**Keywords:** bioenergy crops, *cis*‐regulatory elements, ATAC‐seq (transposase accessible chromatin sequencing), DAP‐seq (DNA‐affinity purification and sequencing), multi‐part constructs, modified/synthetic promoters

## Abstract

Advances in engineering of bioenergy crops were driven over the past years by adapting technological breakthroughs and accelerating conventional applications but also exposed intriguing challenges. New tools revealed rich interconnectivity in the exponentially growing and dynamic ‘big' omics data’ of metabolomes, transcriptomes, and genomes at previously inaccessible magnitude (global, cross‐species, meta‐) and resolution (single cell). Insights enabled fresh hypotheses and stimulated disciplines such as functional genomics with discovery of broad regulatory networks and their determinants, that is, DNA parts, including promoters, regulatory elements, and transcription factors. Their rational design, assembly into increasingly complex blueprints, and installation into diverse chassis is an existing frontier that may benefit from emerging technologies to address bottlenecks. Interweaving nature‐inspired to fully synthetic parts has already allowed building of fine‐tuned regulatory circuits, or new‐to‐nature metabolic routes insulated from the biological context of the chassis species. Similarly, developments and the evolving need for unifying principles in plant transformation and species‐agnostic technologies highlight future opportunities for engineering the next generation of bioenergy plants.

## Significance Statement

Combined advances in biotechnology are now tackling multifaceted engineering challenges for enhanced traits in an equally diverse spectrum of bioenergy species. This review discusses the innovative hotbed at the intersection of the conventional foundation of the field with recently established multi‐disciplinary technologies.

## INTRODUCTION

Plant transformation technologies for editing, delivery, regeneration and selection, implemented for agricultural crops, now provide a template for engineering of bioenergy crops. We introduce here briefly influential research and critical reviews, shaping the context of this article.

The CRISPR‐Cas nuclease toolbox has progressed from a targeted, yet error‐prone mutagen to editors with increased accuracy, such as precise base targeted edits and insertions, to insertions of entire DNA fragments. Proof‐of‐concept studies in structural genes have shown broad applications across mono‐ and dicotyledon crops (Jiang et al., 2022; Vu et al., 2024). Cas‐mediated modification of upstream regulators of gene expression has achieved trait improvement in a limited number of crops (e.g., Zhou et al., 2024). In bioenergy plants, technologies for the identification and characterization of the global regulatory machinery have been deployed and are discussed below.

In addition to editing accuracy, the delivery of editing reagents impacts national regulatory classifications and, with that, societal acceptance for all crops. Technical challenges to generate transgene‐free, heritable gene‐edited agricultural crops were overcome on the level of a few individual species (or genotype) (Kocsisova & Coneva, 2023), but are further compounded in bioenergy species. In those, researchers are confronted with combinations of obstacles, including heterozygosity, polyploidy, self‐incompatibility, perennials with long generation times, and transformation recalcitrance. A recent demonstration bypassed tissue culture, regeneration, delivery, and the need for crosses through a transgenic rootstock to mobilize CRISPR RNAs into a grafted wild‐type receiver (Yang et al., 2023). Yet, this method is inherently limited to bioenergy species with established grafting protocols such as poplar, currently excluding monocot species. In sorghum, delivery and generation of transgene‐free edited lines were achieved by microprojectile‐mediated transformation (Zhang, Cheng, et al., 2025). Despite technical barriers intrinsic of the method, including tedious plant regeneration, it should be compatible with bioenergy crops such as sugarcane, which are vegetatively propagated.

In 2016, a major hurdle was overcome for the transformation of monocotyledon species. The discovery that co‐expression of the maize (*Zea mays*) developmental regulators, the transcription factors Baby boom (BBM) and Wuschel2 (WUS) stimulated both growth of transformable tissue and, importantly, regeneration and recovery of transgenic plants across genotypes (Lowe et al., 2016), heralded a flurry of technical advances. These included transformation of sugarcane and sorghum, next to a broad spectrum of monocot crops. In the wake of the BBM/WUS boom, other novel factors with similar outcomes were discovered, including Growth Regulating Factors (GRFs), WUS ortho‐, homologs, and other developmental regulators, which together expanded accessible explant tissues and are now spanning a broad range of seed plant species, including the bioenergy crops referenced here (Pan et al., 2022; Youngstrom et al., 2025).

**Figure 1:**
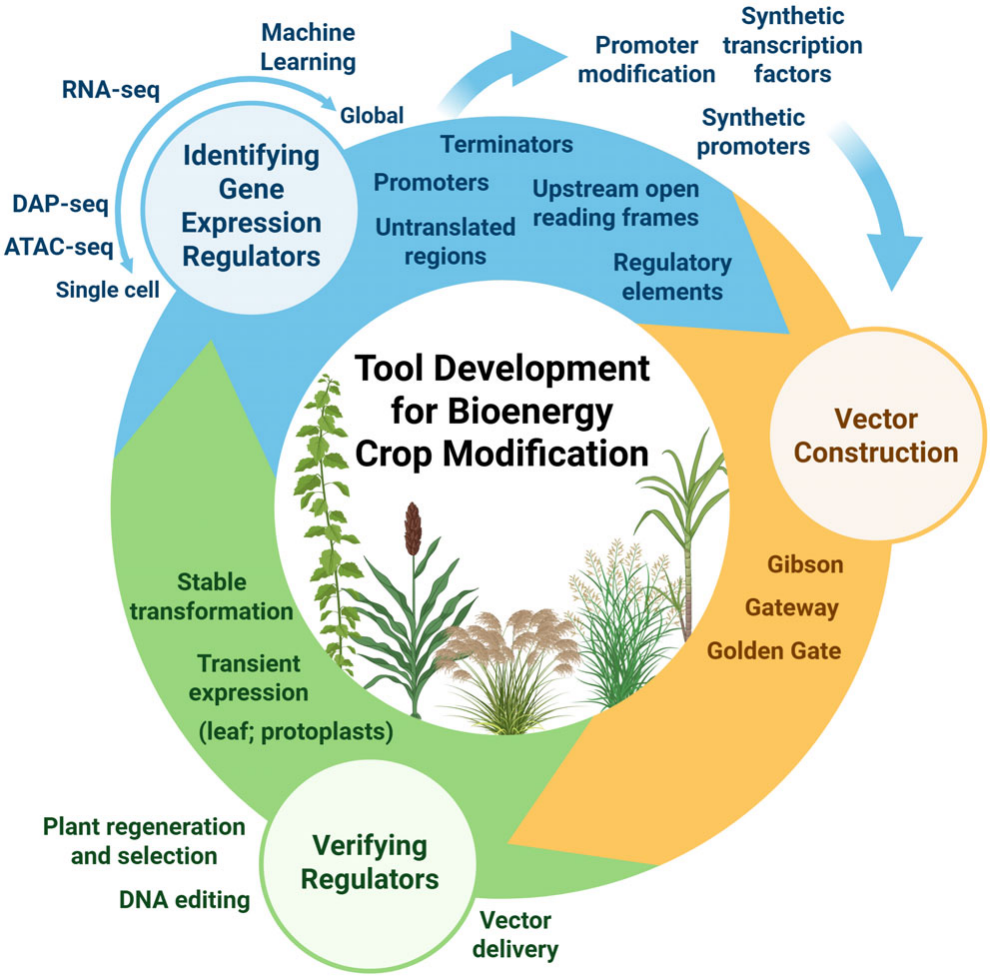
Roadmap of the integrated tools advancing bioenergy crop improvement. Distinct technologies are the foundation for three central concepts discussed in this review, including scaled identification of regulatory parts, their rational modification, design, and assembly into complex multigene cassettes for delivery of the transgene cargo. Validation and transformation further guide refinement of the next generation of parts.

All above, and related recent breakthroughs using viral, or direct in planta gene manipulation (Belaffif et al., 2025; Chamness et al., 2023), represent the methodical foundation for the engineering of bioenergy crops. The raw material, mined and validated gene parts to enable this transfer, is provided by new strategies and advances generating and integrating ‘Big' Omics Data’. This review presents a roadmap (Figure 1) focused on DNA‐based tools, that is, the vital parts making up the blueprint of the transformation cargo built by synthetic biology. Transcriptional regulatory networks reveal promoter candidates that enable spatiotemporal control of expression of structural genes at greatly increased resolution. Technologies facilitating discovery of both elements residing in promoters, regulatory regions, and the transcriptional machinery are reviewed in historical context, but with focus, where applicable, on bioenergy crops. As secondary tools, characteristics of popular vectors are highlighted, delivering the cargo for genetic manipulation in context outside of model organisms. Recent examples for metabolic engineering underscore the central importance of assembly and delivery of a complex and sizeable payload for engineering traits. Hence, we illustrate next to technical progress in the genomic part assembly, practical considerations, including acceleration of design, engineering, and boosting of combinatorial throughput. Consequently, we highlight throughout this work the contributions and broad potential that transient transformation can offer. This includes, despite being tissue‐, or protoplast‐centric, the rapid validation of parts from fast‐growing candidate pools. We finally illustrate that the assembly of genomic parts into synthetic, that is, new‐to‐nature circuits, can enable targeted reprogramming of traits in response to complex input signals.

Innovations are highlighted, where understanding the interplay of genomic regulatory parts, and their assembly into complex units are going hand in hand with efforts to push the envelope of transformation procedures. Together, these technologies allow the building and installation of genetic circuits to control traits and drive the next generations of improved bioenergy crops.

## CONVENTIONAL APPLICATIONS OF GENE REGULATION

### Natural promoter candidates, transcriptional regulatory networks

From a transgene engineering perspective, native promoters and candidates can be classified according to their activity as constitutive, signal‐responsive, and developmental and tissue specific (increasingly cell type specific) based on their transcriptional strength and context constraints.

So‐called constitutive promoters, often derived from pathogens and plant housekeeping genes, are considered non‐regulated tissue and stimulus independent and are typically deployed to drive ectopic expression. Examples such as cauliflower mosaic virus (CaMV) 35S, *Zea mays* (maize) ubiquitin (Ubi), and *Oryza sativa* (rice) actin (Act1) were identified early in plant molecular genetics and remain widely used today (Christensen et al., 1992; McElroy et al., 1990; Odell et al., 1985).

Since the 1980s, native promoters have been studied by linking them to reporter genes like b‐glucuronidase (*GUS*), fluorescent proteins, or luciferase. Significant discoveries were enabled over the past three decades by forward genetics approaches, including promoter trap and gene trap techniques (Galbiati et al., 2008; Topping et al., 1994; Topping & Lindsey, 1997). These methods unveiled novel promoter activities and specificities, facilitating their use as developmental‐, organ‐ or cell‐specific drivers, including in bioenergy crop species, such as those in focus of this review: poplar (*Populus spp*.), switchgrass (*Panicum virgatum*), sugarcane (*Saccharum officinarum*), miscanthus (*Miscanthus* spp.), and sorghum (*Sorghum bicolor*). Building on and expanding our portfolio of cell‐type relevant tools has been accelerated by recent advances in single cell analysis and transcriptomics (scRNA‐seq). An illustrative study in sorghum reported cell‐type specific, spatial transcriptomes from the stem, with tissues isolated by laser capture microdissection (Fu et al., 2024). These data can support identification of plant promoter candidates at unprecedented resolution.

Historically, the Arabidopsis (*Arabidopsis thaliana*) root has served as an example to approach the modulation of gene regulatory networks by transcription factors (TFs), enabled by the lack of autofluorescence background, typically caused by chloroplast photopigments in many aerial tissues. Fluorescence driven by promoter‐GFP constructs for 61 root‐related TFs was compared with mRNA expression data (Lee et al., 2006). Sequences or elements residing within 3 kb of the upstream non‐coding region for four out of five candidates were found sufficient to drive fluorescence consistent with the expression data. The principle will be further discussed in the next section. For the remaining candidate, indications were found for regulation at post‐transcriptional level, with yet unknown elements proposed to reside in the mRNA (Lee et al., 2006). This work was expanded from the limited number of reported tissue types to a more comprehensive set of 14 non‐overlapping root cell types and subsets of characteristic expression patterns for individual genes (Brady et al., 2007; Marquès‐Bueno et al., 2016).

This and related studies provided a template for building of collections of cell‐type specific promoters and marker lines, as tools for a spectrum of downstream applications and the growing community with focus on functional genomics (Marquès‐Bueno et al., 2016). The regulatory networks controlling spatiotemporal responses, for example, hormonal‐mediated developmental transitions, remain an intensely researched area (Nolan et al., 2023). For example, the drought inducible *ZmRAB17M* promoter was used to drive the CRE/loxP system in sorghum for recombinase‐based removal of *WUS2* and *BBM* after embryogenesis and stable integration of transgenes before plant regeneration (Mookkan et al., 2017). The *phosphoenolpyruvate carboxylase* (PEPC) promoter was used to target ZmWRI1 to the mesophyll for increased production of lipids in sorghum leaves (Vanhercke et al., 2019). The rationale for this promoter choice was to drive transgene expression under light conditions, constrained to the mesophyll cells where PEPC is the initial acceptor of carbon in C_4_ plants. *PEPC*‐driven transgenics contained higher palmitic acid (C16:0) levels compared to *ZmWRI1* driven by the *ZmUBI* promoter, which had increased linoleic acid (C18:2) levels. This shift in profile supports a cell‐type responsive component of lipid metabolism. Lipid production in sorghum is expanded upon below. Despite these advances of the field, identifying candidates and regions upstream of the translational start site (TSS) remain the foundation for annotation of individual *cis*‐regulatory elements (CREs) and, importantly, their comprehensive functional validation.

### *Cis*‐regulatory elements

*Cis*‐regulatory elements are typically short DNA sequences that modulate gene expression via their interaction with *trans*‐acting factors, including TFs or chromatin remodeling factors (Wolter & Puchta, 2018). TFs that bind to CREs either activate or repress transcription of the gene by interacting with other TFs and/or transcriptional machinery through their effector domains (Hummel et al., 2023).

The promoter and the 5′ untranslated region (UTR) characteristically carry CREs, considered primarily responsible for gene expression as the site for transcription initiation and polymerase II binding (Brooks et al., 2023). Identification of CREs responsible for tissue‐ and development‐specific gene expression can enable specific, targeted gene expression for engineering bioenergy crops. The discovery that genes encoding enzymes of the phenylpropanoid pathway are readily induced by UV light or pathogen factors set the stage in the late 80s for a new era of promoter dissection and discovery of CREs (Lois et al., 1989). Scientific breakthroughs included *in vivo* DNA footprints: the mapping of regions protected by proteins (the footprint) from DNase I cleavage. Thus, short promoter regions necessary for inducibility and sufficient for developmental expression could be defined (Schulze‐Lefert et al., 1989), inspiring their testing in poplar (Gray‐Mitsumune et al., 1999). Challenges encountered then remain today, including occasional discrepancy of promoter–reporter detection and gene expression. This triggered the discovery of CREs in introns, the 3'UTR, and terminator, as well as in distal sites, which illustrate the complexity of regulation and highlight challenges for accurate reconstruction for biotechnology (Douglas et al., 1991).

One of the first high‐throughput methods for the genome‐wide, unbiased identification of TF binding sites applies DNA‐affinity purification and sequencing (DAP‐seq) using expressed and purified TFs with libraries built from genomic DNA. This technology emerged for plants in 2017 and surpasses the earlier used chromatin immunoprecipitation for both economics and scalability (Bartlett et al., 2017). JGI (U.S. Department of Energy Office of Science user facility, Joint Genome Institute, Lawrence Berkeley National Lab) published a revised protocol in November 2021, streamlining the workflow. Despite resolving bottlenecks such as the need for the labor‐intensive purification of *in vitro* translated TFs, challenges remain, including optimization for specific families and lack of potential post‐translational modifications not accommodated by the non‐native processing. Even so, this JGI high‐throughput capability is now implemented as part of JGI's Community Service Program (CSP; https://jgi.doe.gov/user‐programs/program‐info/csp‐overview/) and bioenergy species are recognized as the new frontier. Examples in sorghum and poplar highlight the power of the approach. In collaboration with the JGI, RNA‐seq and DAP‐seq led to the characterization of a poplar master regulator in plant‐endosymbiont (ectomycorrhizal symbiosis) interaction. The TF (MYC2, boosting directly jasmonic acid (JA) responsive genes) was found to specifically mediate a complex choreography of other transcriptional response and hormonal cascades. Those culminate, among JA responses, in triggering of terpene biosynthetic genes, and with that impairing of fungal colonization (Marqués‐Gálvez et al., 2024). Fontanet‐Manzaneque and coworkers used DAP‐seq in sorghum to demonstrate that a TF (SbBES1, removing suppression of brassinosteroid (BR) driven muting of drought response) binds to a specific motif and mediates the BR homeostasis‐controlled drought adaptation through activation of the flavonoid route (Fontanet‐Manzaneque et al., 2024).

Many new methods have been developed to increase the resolution from DNA regions to the identification of individual CREs for specific genes. Databases of conserved elements, such as from Plant Transcription Factor Database (PTFDB 4.0; Jin et al., 2017), Plant Promoter Analysis Navigator (PlantPAN 4.0; Chow et al., 2024), and GRASSIUS (Gray et al., 2024), can inform the identification of potential CREs for genes expressed in specific tissues or during developmental stages. Regions of genomic DNA free of chromatin occupancy are indicative of potential for recruitment to yet unidentified CREs of transcriptional co‐regulators, that is, TFs driving the expression of adjacent genes. Those regions can be identified through assays with a DNA‐cleaving transposase acting on non‐complexed DNA, and sequencing of the non‐cleaved protected DNA. Such Assays for Transposase Accessible Chromatin sequencing (ATAC‐seq) across 13 angiosperm species identified tens of thousands of regions, predominantly adjacent to promoters and in terminator regions, but also distal to genes (Lu et al., 2019). Sorghum and poplar are among the bioenergy species adopted for ATAC‐seq; profiling of the accessible chromatin regions revealed a sharp peak around the TSS (transcription start site), which correlated with enhanced transcriptional activity of the proximal genes (Zhou et al., 2021). Recent advances in massive multiplex capabilities at single cell or single‐nucleus resolution, such as sifi‐ATAC‐seq (Zhang et al., 2024) and multiomics approaches (Zhang, Luo, et al., 2025), could be the next stage of CRE discovery in bioenergy feedstock species.

Without prior knowledge of chromatin accessible regions or global TF expression libraries, machine learning (ML), an artificial intelligence (AI) approach, can assist in identifying potential regions carrying putative CREs. This method utilizes global expression data covering two classification groups (positive and negative) for pre‐selected criteria for training and testing the model, before deploying the algorithm on new data sets. In switchgrass, an elegant study using k‐mer sequences (short string of nucleotides) enriched in the genic and flanking regions of cold‐responsive genes, but not in non‐responsive genes, has identified such condition‐specific CREs. This strategy can prove instrumental in both predicting feedback as well as the discovery of novel elements (Ranaweera et al., 2022).

In the omics era, when CREs are being mapped at genome scale across a growing number of plant species, experimental validation continues to be a bottleneck. Understanding the interactions among distinct and perhaps distally located CREs will also be important for fine tuning transcriptional regulation.

### Validation of *cis*‐regulatory elements

Stable transformation of many bioenergy crops remains an arduous and time‐consuming task. Recent advances in plant transformation and editing technologies (Parrott et al., 2024) are now allowing commercial facilities to offer stable transformation services for the bioenergy crops switchgrass, sorghum, and poplar highlighted in this article, including the Danforth Plant Science Center and the Universities of Missouri, Rhode Island, Wisconsin‐Madison, and Texas Tech, all based in the United States.

Establishing a method for transient expression in bioenergy crops via infiltration can allow for expedited testing of regulatory elements before investing in stable transformants. Transient expression via *Agrobacterium tumefaciens* infiltration is a robust routine in the dicot *N. benthamiana* and is commonly used for heterologous expression. Even though the principle of transient transformation and expression was demonstrated for poplar, switchgrass, and sugarcane, results were focused on regeneration of plants from a limited set of cellular events. In sorghum, recent improvements were reported in protoplast isolation and transformation (Lee et al., 2023; Meng et al., 2020). Those must conquer physiological and molecular challenges as seen in other species. For example, while cell‐type identity was retained for at least 48 h in petunia (Faraco et al., 2011), stress from protoplast preparation can impact chromatin structure, with genome‐wide increases in chromatin accessibility altering gene expression (Probst & Mittelsten Scheid, 2015; Reyna‐Llorens et al., 2023). This confounds cell‐type identification by marker gene expression (Sawers et al., 2007) and likely impacts accurate CRE characterization. Marking a potential critical breakthrough, the first two studies demonstrated transient expression in sorghum leaves via agrobacterium infiltration (Miller et al., 2023; Sharma et al., 2020). To improve efficiency, however, transient sorghum expression will require further multi‐factor optimization to build a much‐needed community‐shared open‐source procedure, sufficiently robust for high‐throughput characterization of regulatory elements. Following suit, complementary demonstrations in poplar and switchgrass, as well as development of the respective tools are urgently being looked‐for.

### Vector systems for bioenergy crop engineering

The earliest reports of applications using T‐DNA binary vectors and disarmed Agrobacterium strains date back four decades (Hoekema et al., 1984), despite a very narrow plant host range at that time. Based on their now expanded use and illustrative technologies for cloning, which became increasingly complex as the community moved to larger constructs with multiple expression cassettes, we are highlighting different binary vector systems and their characteristics with a focus on bioenergy crops. All tools are readily available to academic and non‐profit scientists under a Uniform Biological Material Transfer Agreement (UBMTA, Addgene, Watertown, MA, USA).

Cambia (Canberra, ACT, AU, CAMBIA.org) is a Non‐Governmental, Non‐Profit Organization established over 30 years ago and ranked as one of the world's top NGOs in 2013. While their Biological Open Source was launched in 2005, the first work citing their diverse line of legacy vectors for effective plant transformation dates to 2001 (Zheng et al., 2001), with reports in the bioenergy crops sugarcane, poplar, switchgrass, and maize emerging in the next decade (Chen et al., 2010; Groover et al., 2004; Han et al., 2009; Mayavan et al., 2013). The pCAMBIA vectors are widely popular for their flexibility and modularity, stability, high copy number in *E. coli*, and, at approximately 8.5 kb, moderate size for an empty binary vector backbone. An extensive variety of adaptations have been made and shared by the community, including simple constructs for *in planta* promoter testing with a range of reporters, expression in novel hosts including plant‐associated microbes, and implementation of site‐specific recombination without the need for restriction enzymes and ligases. While early pCAMBIA variants for genome editing have been developed and validated in maize (Xing et al., 2014), recent advances in CRISPR‐based multiplex genome editing in bioenergy crops have targeted largely expanded tandem arrays of highly similar genes (Bewg et al., 2022; Chen et al., 2023), or elegantly up to six genes involved in lignin biosynthesis simultaneously (Sulis et al., 2023). Currently, over 400 pCAMBIA variants are available to the community (addgene.org), reflecting that these vectors are flexible, open‐source workhorses for routine plant transformation to deliver relatively simple cargo into a limited range of plant species.

Beyond vector systems, the open‐source philosophy and initiative for sharing of plant transformation tools of Cambia is laudable (Biological Innovation for Open Society, further reading at https://cambia.org/bios‐landing/the‐cambia‐bios‐initiative/, accessed 01/2025). New technologies have been developed to bypass critical IP/license restrictions in plant engineering, such as gene transfer by Agrobacterium or screening for transgene behavior. Outstanding examples include the development of the biological gene transfer system for eukaryotic cells (TransBacter™), which uses a species outside the genus Agrobacterium. Compatible with this system is a β‐glucuronidase reporter GusPlus™ with sensitivity exceeding the *E. coli* GUS but derived from *Staphylococcus*. Additional optimized features were developed as resources for the community (cambia.org). Yet, pCAMBIA vectors are not without potential problems; because in some backbones, both the gene of interest and the selectable marker are driven by the double CaMV35S promoter, trait stacking involving re‐transformation using the pCAMBIA backbone led to co‐suppression in advanced generation of (homozygous) Arabidopsis (Ortega et al., 2024). Co‐suppression or gene silencing of this nature is less of a problem in transient expression, especially when the silencing suppressor P19 from the tomato bushy stunt virus is also co‐expressed (see pEAQ‐vector below). This factor binds and sequesters double stranded RNAs, effectively inhibiting the post‐translational gene silencing machinery (Silhavy et al., 2002). It was reported that CaMV35S can compromise expression outside of its 3′ downstream target, namely of the reporter in the construct. This interference disappeared without the strong activator in pCAMBIA and other related vectors (Yoo et al., 2005). A work‐around suggested by Cambia and later demonstrated as feasible with reasonable frequencies in crop engineering, relies on co‐transformation of independent T‐DNA, one for the marker and the other for the transgenic trait. This crop transformation approach can conveniently afford marker free plant lines at the same time (Liu et al., 2020). An alternative practice more amenable for engineering and synthetic biology was demonstrated recently, when short insulator‐like elements, found in the minimalist genome of *Utricularia gibba* (bladderwort), efficiently suppressed read‐trough in multi‐module cassettes (Laspisa et al., 2023).

Original vectors still in use for poplar date back four decades, when a versatile, relatively small, stable, and fully sequenced plasmid was reported (Bevan, 1984). This vector represents an example of how delivery tools were adapted over the years with several of the above technologies to improve efficiency in the engineering of bioenergy crop species.

Two vector systems were developed, taking advantage of the Gateway system (see next section below), to facilitate high‐throughput cloning and accommodate the increasing needs for complex construct assembly. In monocot species, the pANIC vector system demonstrated versatility in functional overexpression and gene suppression in switchgrass, rice, and a range of species such as *Brachypodium distachyon* and sorghum (Mann et al., 2012). A widely used and continuously adapted, derivatized, and improved vector system for dicotyledon plants is pEAQ, or its sibling pEAQ‐*HT* (Easy And Quick‐Hyper Translatable), developed by the team of George Lomonossoff (Sainsbury). Overall moderate size (e.g., 7.3 kb for pEAQ‐GFP‐*HT*), combined with a module for suppression of transgene silencing (P19, from tomato bushy stunt virus), and a modified 5′ and 3′UTR of CPMV RNA‐2 flanking the plant expression cassette for expression enhancement, are attractive features (Peyret et al., 2019). An inherent advantage is the plant co‐infiltration with *Agrobacterium* strains independently harboring single genes per individual plasmid for transient expression. This strategy permits the combinatorial *in planta* assembly of complex multi‐step pathways, including controlled subcellular localization to take advantage of native compartmentalization (Bibik et al., 2022; Bibik et al., 2024a). A heavily modified pEAQ‐*HT* vector allowed delivery of multigene constructs into poplar affording plastidial formation and accumulation of high‐value bioproducts in leaves (Bibik et al., 2024a; Bibik et al., 2024b). The MTA for pEAQ and its derivatives is available through Plant Bioscience Limited, Norwich Research Park, UK.

### Cloning, assembly, and delivery of multi‐part constructs

Precision engineering of bioenergy species to reduce recalcitrance towards extraction and conversion of cellulosic biomass and digestibility of lignin is well established (reviewed in Ning et al., 2021). Examples include cell wall directed traits in poplar ‘ZIP’‐lignin, or switchgrass modified exclusively in above‐ground tissues, enhancing bioenergy yields (Liu et al., 2018; Unda et al., 2022, 2024). Metabolic engineering for value‐added bioproducts has been reported generally, including commercial considerations (reviewed in Lange & Ahkami, 2013). Yet, for bioenergy crops this strategy remains in its infancy, with limited demonstrations (Bibik et al., 2024a; Bibik et al., 2024b; Park et al., 2024). Coproduction of high‐value compounds could offset biofuel production costs and improve competition with petrochemistry by providing alternative access to bioproducts. In addition to the socioeconomic impact reduced carbon emission and pollution can have for vulnerable populations, wide implementation of bioenergy crops such as sorghum can be of timely ecological relevance, in part due to its extensive root system (Ngidi et al., 2024). Aiding its resiliency for growth on land and at water restrictions unsuitable for food crops, sorghum can reduce erosion and improve soil health through sequestration of organic matter and (Dignac et al., 2017).

Conquering both traits of enhanced cellulosic biomass and novel bioproducts in one ‘multi‐use’ chassis requires the assembly of platoons of DNA parts and their successful deployment. In recent years, bulk synthesis of DNA building blocks has become affordable due to advancements in automation, chemistry, and competition. Assembly of DNA parts (promoters, UTRs, coding sequences, terminators, etc.) into initial transcription units (assembled DNA expressing one, or more genes), then into multigene constructs (defining a complex trait) was supported by new tools (e.g., Chamness et al., 2023; Patron et al., 2015; Vazquez‐Vilar et al., 2021), including solving the puzzle of short repetitive AT‐rich sequences, with solutions bypassing limitations of conventional DNA synthesis (Fernandez‐Moreno et al., 2024). Each of the assembly technologies has their own intricacies and the choice depends on a variety of factors, prescribed by the experimental, and user needs. For a comprehensive overview, the reader is referred to selected reviews and primary articles cited in the following brief overview. Parts, tools, plasmids, where available as open source, or via Addgene are referenced as starting point.

The early BioBrick™ standard describes strict physical definitions of DNA parts to conform to the open‐source Registry of Standard Biological Parts, now carrying over 20 000 documented parts (iGEM). The rules, which were introduced in 2003 (Knight, 2003), include removal of illegal restrictions sites (‘domestication’) and compatibility with Type IIS assembly, that is, restriction enzyme sites with offsite cutting by endonucleases cleaving DNA distal (4–8 nucleotides) to their recognition site. By now, several assembly methods for BioBricks™ are supported, including overlap‐dependent, ligation‐free assembly and Type IIS compatible sections of the modular cloning principle (e.g., Gibson and MoClo; see below). The BioBricks Foundation freely distributes an additional 10 000 parts (biobricks.org) and notably led the collaborative effort with the former OpenPlant initiative towards open options for material transfer agreements, simplifying universal sharing of biological parts (OpenMTA, Kahl et al., 2018). Still based on Type IIS enzymes, the cloning strategy ‘Golden Gate’ offers one tube, one step‐built recombinant expression vectors from an entry clone, through user pre‐defined 4 nucleotide overhangs, and hence became scarless (Engler et al., 2008). Researchers have since then continued to contribute to the shared parts, with Addgene currently offering over 300 vectors and parts related to Golden Gate plant constructs. Addgene early adopted the Uniform Biological Material Transfer Agreement (UBMTA), initiated in 1995 by the National Institutes of Health (NIH) and covering most parts. With the notion that the transferred material may be subject to patent protection and can be used for patented applications, the UBMTA limits transfer to academic institutions and other non‐profit organizations.

The Gateway™ system (former Invitrogen, now Thermo Fisher Scientific) is a cloning technique that uses specific recombination sites and proprietary enzyme mixes to transfer parts between entry vectors and expression vectors. Of the collection of about 800 plant Gateway™ vector variants which are available at Addgene, the popular pEarleyGate series was found compatible across mono‐ and dicot species (Earley et al., 2006) and provided the foundation for the next iteration of switchgrass transformation vectors (pANIC, see above).

Overlap‐based cloning poses the fewest sequence‐based constraints. Both prevalent Gibson® (New England Biolabs) and In‐Fusion® (Takara) methods employ exonucleases to generate DNA with 3′ or 5′ overhangs, respectively, for the plasmid and insert. Complementary overhangs of the insert, introduced through primers by PCR, anneal with vector overhangs and joined into scarless plasmids. Overlap‐based generation of complex, multigene constructs with In‐Fusion® has proven practical for assembly of large pEAQ‐based constructs for stable transformation of poplar, as well as rapid library building for transient combinatorial testing.

The acronym MoClo (modular cloning) stands for an organizational definition relying on the principle of directional and hierarchical multi‐part assembly. Parts are migrating from ‘level 0’ plasmids (termed Phytobricks) via assembly into ‘level 1’ modules of functional transcriptional units, before combination in ‘level 2’ binary vectors for transformation. Examples are compatible with most assembly methods described above, but common in plant transformation is Golden Gate. GoldenBraid (2.0), an iteration specifically dedicated to plant engineering combines the features of an exchangeable parts collection, a positional cloning scheme, with available web resources for domestication and engineering (Sarrion‐Perdigones et al., 2013). An excellent comparison of the principles, and for the newcomer much‐needed simplification of exceedingly complex procedures, is given by Chamness and coworkers (Chamness et al., 2023). Notably in the context of this review, regulatory elements compatible with mono‐ and dicot expression, promoters and terminators are tested, as well as in part unspecific long‐distance effects of enhancer elements are examined (Chamness et al., 2023).

Bioenergy crops building up energy‐dense lipids are aiming at contributions to the large markets for transportation fuels. The combinatorial co‐expression of a set of three genes in the inbred sorghum grain variety Tx430 achieved over 8% in leaf dry weight of triacylglycerol (‘vegetable oil’). While not reaching the ultimate target of 15% per DW to compete with, for example, oil palm, the study demonstrated the implementation of a popular multipronged strategy, termed ‘push‐pull‐protect’ in a bioenergy crop (Vanhercke et al., 2019). The three key factors, with homologs derived from several plants in different studies with similar results, demonstrate a cooperative principle: together, a TF master regulator of lipid formation in plastids, WRINKLED1 (WRI1), a biosynthetic enzyme fixing the fatty acids onto the glycerol backbone diacylglycerol acyltransferase (DGAT) and a protein stabilizing the emerging lipid droplets Oleosin (OLE) are deployed in transgenic bioenergy crops. Biomass and fitness in sugarcane, excessive gene copy numbers, and problematic genetic stability in sorghum were identified bottlenecks. A follow‐up study changed from biolistic to agrobacterium‐mediated transformation and installed in addition two lipid metabolic enzymes (Park et al., 2024). The cargo for transformation consisted of up to six transcriptional units, including selection marker, *WRI1/DGAT/OLE* and two auxiliary genes, driven by a palette of common constitutive (UBI/35S/PGD (phosphogluconate dehydrogenase)) promoters and two WRI1‐responsive synthetic elements, assembled by GoldenBraid. This study is noteworthy for (i) demonstration of heritability, a precondition for introgression into agriculturally relevant hybrid varieties, (ii) reported oil concentrations exceeding 5% of DW from field‐grown plants without impact on biomass over two seasons, and (iii) a stable chemotype over several generations (Park et al., 2024). Capitalizing on the need to manipulate multiple gene targets (i.e., *WRI1, DGAT, OLE*), high throughput and a testing system to scale transformations, a prototyping pipeline was developed in maize and *N. benthamiana* (Dong et al., 2025). This study automated Golden Gate cloning, and integrated protoplast and callus cell transformation with targeted analytics at single cell scale. As such, this approach could provide future traction for transgene engineering supported by biofoundry services compatible with plant engineering (‘NSF Invests in BioFoundries to Drive Advances across Science and Engineering, NSF—National Science Foundation’ 2024).

All these technologies capitalize on the assembly of large multi‐part constructs, yet still at the cost of the ease of cloning and yield of the resulting oversized plasmids. Reducing the size of the cargo for multigene delivery can take advantage of a reduction in part numbers, specifically the promoter regions and numbers. Driving expression of two coding regions on a single mRNA and processing of the chimeric protein can afford two discrete functional polypeptides. The individual parts of a hybrid linker were discovered independently yet complement each other. The first, the LP4 peptide, originates from a natural polyprotein occurring in the seed of garden balsam (*Impatiens balsamina*), which is split between the first and second amino acids in post‐translational processing. The second, derived from the foot‐and‐mouth‐disease virus (FMDV) 2A sequence, is co‐translationally cut at its C‐terminus. The *combined* action of both removes nearly the entire amino acid linker (François et al., 2004). This feature is important for the second protein which may carry targeting sequences such as a plastidial N‐terminal tag (Bibik et al., 2024a). Two points are noteworthy from a practical perspective. First, the efficiency of cleavage in a chain of fusions is typically reduced beyond practical applications after two proteins. Secondly, and following parts such as the *35S CaMV* promoter and the *P19* suppressor, the use of elements of viral (pathogenic) origin in constructs significantly complicates regulatory approval.

Bidirectional promoters, simultaneously regulating expression of genes on both ends, indeed reduce the number of regulatory parts and reduce the risks of repetition of single promoters. In yeast engineering, they were early established, for example, the galactose inducible ←GAL1/GAL10 → element and development in other microbes for dual‐gene co‐expression followed (Vogl et al., 2018). Analogous tools in plants are under‐studied, despite an early proof‐of‐concept with constitutive tail‐to‐tail fused promoters (Xie et al., 2001). Comparative genome analysis between poplar and other angiosperms suggested diverse mechanisms for shaping convergent gene pairs across species (Krom & Ramakrishna, 2008). In rice, promoter bashing of intergenic regions identified first the enhancing region which sharply increased its bidirectional expression efficiency and secondly the essential regions respectively responsible for its 5′ and 3′ basic expression activity (Wang et al., 2016). Recently, a largely bioinformatic study investigated potential bidirectional promoters in the crop cotton (*Gossypium hirsutum*) (Yang et al., 2024). Transient expression of reporter constructs indicated that native, genomics‐informed mining of functional, tissue‐specific regulatory elements could significantly benefit from integration of technologies such as ML (see above) for *de novo* discovery in bioenergy species.

While the T‐DNA *delivery* into the plant (natively at 200 kb+, Ti‐plasmid) appears to pose no size limitation, a challenge comes from particularly bulky T‐DNA and their bacterial *maintenance* (Song et al., 2003). The efficiency of plant integration of intact T‐DNA was improved through several approaches. Empirical evidence shows degradation of the T‐DNA at the 3′‐end or toward the left border. To increase the frequency of recovered events with intact T‐DNA, marker genes located at both borders were suggested (Collier et al., 2018). Noteworthy is also an increase by up to 100% in stable plant transformation efficiencies by modified origins of replication, achieving an increased copy number of the binary vector (Szarzanowicz et al., 2024).

The genomic stability of the T‐DNA insert itself is conveniently tractable by, for example, genotyping. Only recently, with the advances in genome resequencing of transgenic events, their detailed characterization on a genomic level has become accessible to the broader community. These pitfalls have recently been the subject of a comprehensive review and will only be mentioned here (Raabe et al., 2024). In brief, T‐DNA insertion can have a profound impact on the structural integrity of the locus and the entire plant genome. Multifaceted consequences can confound the interpretation of the plant phenotype and the heritability of individual events. The insertion may lead to immediate alterations of the plant locus, including unwanted insertions, deletions, and multi‐copy events. The latter may impact genetic stability and predictable inheritance. Integrations may also give rise to translocations, genomic rearrangements, epigenetic effects, and background mutations (Raabe et al., 2024).

## NON‐NATURAL TO SYNTHETIC CIRCUITS AND ORTHOGONAL PATHWAYS

### Opportunities for synthetic genetic circuits for dynamic gene regulation

Past functional genetic research has revealed the identity of key genes responsible for most of the valuable traits. In the bioenergy field, representative parts include rate‐limiting biosynthetic enzymes and stress‐responsive regulators, such as those to abscisic acid. Following five decades of discovery in model systems, and translation into crops, defining the rise of plant molecular biology (Somssich, 2022), many valuable agronomic traits in crops have been studied, and their responsible genes were identified; however, translating these fundamental gene discoveries into functional crop improvements is limited by a lack of precise gene regulation tools. Conventional approaches like constitutive overexpression and mutation can cause pleiotropic trade‐offs—where expressing a gene enhances one trait but negatively impacts others due to its activity, or non‐physiologically high expression across multiple tissues and stages. Synthetic circuit systems offer a solution by enabling dynamic and precise gene regulation. Here we focus on synthetic circuits at the transcriptional level. Other synthetic regulation systems, such as those targeting RNA, translation, and protein stability, have been primarily developed in microbes and mammalian cells but require further testing in plants (English et al., 2021; Gao et al., 2023).

**Figure 2:**
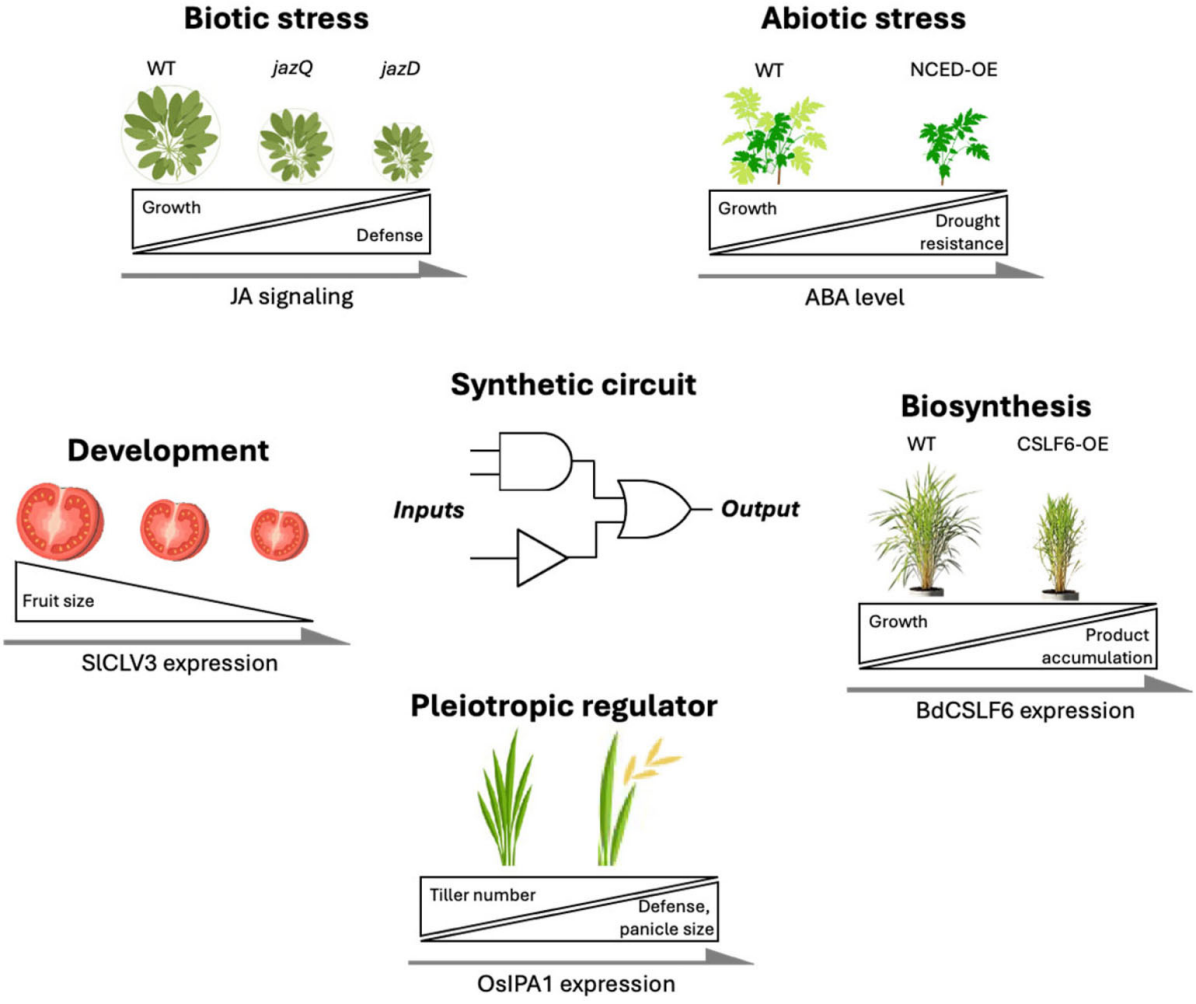
Synthetic circuit for dynamic gene regulation in plants. In biotic stress, the Arabidopsis JA‐MYC transcriptional module balances growth and insect resistance (Guo et al., 2018). For abiotic stress, the ABA‐abscisic acid responsive element module in Arabidopsis manages the balance between growth and drought resistance (Iuchi et al., 2001). In metabolic engineering, constitutive expression of Brachypodium *CSLF6* leads to overaccumulation of mixed‐linkage (1,3;1,4)‐β‐glucan, compromising growth (Kim et al., 2018). In development, tomato fruit size is linked to the expression level of *SlCLV3* (Rodríguez‐Leal et al., 2017). Additionally, pleiotropic genes like rice *OsIPA1* respond to multiple stimuli and regulate several key agronomic traits.

Trade‐offs are a common issue in plant breeding (Figure 2). Metabolic engineering, for example, aims to boost the production of valuable compounds by increasing the expression of key enzymes and regulators. However, overexpressing an enzyme can lead to feedback regulation, disruption of other pathways, toxic byproducts, and inefficient energy use. For instance, constitutive expression of the cellulose synthase‐like F‐family member CSLF6 enzyme caused an overaccumulation of mixed‐linkage glucan (MLG) in the cell wall, leading to compromised growth (Kim et al., 2018; Vega‐Sánchez et al., 2015). A series of Arabidopsis transgenic lines expressing the rice *OsCSLF6* showed a correlation between increased levels of MLG and smaller plants with chlorotic leaves, while transient expression of *OsCSLF6* in tobacco leaves caused severe leaf necrosis (Vega‐Sánchez et al., 2015). Transformation of the model cereal barley (*Hordeum vulgare*) with *HvCSLF6* led to either lethality or smaller plants with leaf necrosis (Burton et al., 2011). Another attempt in the bioenergy model species *Brachypodium distachyon* by expressing the CSLF6‐enhancing transcription factor *BdTHX1* led to stunted seedling development (Fan et al., 2022). A shared feature of all these constructs is the use of unregulated promoters. Similarly, the above‐referenced strategy overexpressing three coordinately functioning lipogenic factors (Wrinkled, WRI1; Diacylglycerol acyltransferase, DGAT1, and Oleosin, OLE1) increased lipid accumulation but resulted in stunted growth and reduced biomass across species (Cao et al., 2023; Parajuli et al., 2020; Vanhercke et al., 2019).

In the context of biotic stress, knocking out susceptibility genes or overexpressing resistance genes often improves pathogen resistance but can lead to growth penalties (He et al., 2022). A well‐known example is jasmonic acid (JA) signaling, which mediates growth‐defense trade‐offs by regulating transcriptional factors (Jasmonate ZIM domain repressor, JAZ; TF of superfamily related to *Myelocytomatosis avian virus oncogene*, *MYC*) and inhibiting growth‐promoting hormones in response to herbivore wounding (Figure 2) (Guo et al., 2018; Guo et al., 2022). As a result, alleles that confer resistance without significant trade‐offs are rare and highly valuable (Qi et al., 2024; Deng et al., 2017; Wang et al., 2022; Sha et al., 2023). For abiotic stresses like drought and heat, overexpression of the hormone and signaling molecule abscisic acid (ABA) biosynthesis, ABA receptors, and ABA‐responsive genes with ABA‐responsive elements in their promoters could improve water‐use efficiency and stress survival but causes growth penalties under non‐stress conditions (Iuchi et al., 2001). In development, traits such as tomato fruit size correlate with expression levels of a small protein signal of the CLAVATA signaling pathway (‘club‐like’, *SlCLV3*) (Rodríguez‐Leal et al., 2017) (Figure 1). Some key breeding targets are highly pleiotropic, governing multiple agronomic traits. For example, increased expression of rice TF *Ideal Plant Architecture* (*OsIPA1*) improves plant architecture but reduces panicle number (Wang & Wang, 2017). Similarly, the Green Revolution's semi‐dwarfing mutations in the *Reduced height* (*Rht*) genes in wheat enhanced lodging resistance but reduced nitrogen use efficiency (Liu et al., 2022).

These examples highlight the need for a targeted reprogramming of gene expression to integrate environmental and developmental inputs, make logic decisions, and regulate output genes to influence various aspects of plant life without compromising key developmental and productivity traits. Such reprogramming is achievable through synthetic circuits.

### Promoter modification and building promoters inspired by native elements

Promoters from well‐characterized genes identified in specific tissues or developmental stages enable targeted gene expression. Indeed, examples demonstrated metabolic engineering efficacy and specificity exceeding viral promoters that are ineffective in vascular tissues, as shown with a lignin‐associated promoter in poplar modulating monolignol composition (Franke et al., 2000). Many genes, however, do not have well‐defined promoters. Traditionally, promoters were based on arbitrary lengths (i.e., typically between 1 and 2 kb), practically informed by cloning strategy (restriction sites) or suitable primer binding sites (GC content). Yet, once sequence‐verified and functionally established, these building blocks represent archetypical parts still shared across labs as physical clones. A prominent example is the 4.8 kb fragment of the tomato polygalacturonase promoter controlling natively fruit ripening‐specific expression. As an engineered part, it drives strong expression with high spatiotemporal control in the same organ (Fernandez et al., 2009). Discovered in tissues undergoing lignification and secondary wall formation, prototypes of core phenylpropanoid pathway promoters, for example, 2.9 kb fragment upstream of the Arabidopsis *cinnamate‐4‐hydroxylase*, and the 2.5 kb promoter from the Eucalyptus *cinnamyl alcohol dehydrogenase*, proved valuable in mimicking the native expression patterns. From a species‐agnostic perspective, the latter two, in instances tested, were active across dicot species, including poplar (Feuillet et al., 1995; Franke et al., 2000). For downstream engineering, determining the shortest regions necessary and sufficient for targeted expression is appealing to reduce the size of constructs to improve efficiency of transformation. In addition, for more complex multigene delivery, a palette of promoters co‐expressing their cargo is desirable for genomic stability and to reduce the risk of transgene silencing. Transgene expression levels are known to be compromised by repetitive, viral, or strong constitutive promoters (Vaucheret & Fagard, 2001). CRE identification can also inform editing of native promoters and the creation of chimeric or synthetic promoters (Aysha et al., 2018). However, experimental characterization remains a bottleneck, especially for tissue types or stress responses that are inaccessible through transient expression. Even with the needed advancement of robust methods for transient expression for the species discussed here (see section on ‘Validation of cis‐regulatory elements’ for current progress), applications will remain limited to the specific tissue type amenable to the experimental approach. The development of CRISPR‐derived technologies has enabled precise promoter editing, minimizing overall genomic modifications in plants (Shi *et al*., 2024). Natural promoter activity is typically governed by multiple CREs, and editing these CREs can directly influence traits. For instance, modifying pathogen effector binding sites in the promoters of genes encoding sugar transporters (SWEET, Sugars Will Eventually be Exported Transporter) in rice confers resistance to bacterial blight (Oliva et al., 2019). Similarly, gradually altering a CRE (CC(A/T)_6_GG, or CArG element) in the tomato *WUS* promoter leads to gradually upregulated *SlWUS* gene expression and thereby proportionately increased tomato fruit size (Rodríguez‐Leal et al., 2017). However, predicting and characterizing CREs can be challenging and time‐consuming. Even with known CREs, mutating a single element may not always yield the desired outcome. For less understood CREs, promoter tiling and screening can help. For example, this approach identified a mutant in a gene that restricts expression of *IPA1* to specific cell types, uncoupling the growth‐defense trade‐off in rice (Song *et al*., 2022). Additionally, CRISPR‐based promoter bashing of the *SlCLV3* promoter produced a range of tomato mutants with a wide spectrum of phenotypic variations in fruit size (Aguirre et al., 2023).

Interestingly, genome editing and screening can uncover unexpected ways to reprogram gene expression. In wheat, mutagenesis targeting the susceptibility gene *TaMLO1* (ortholog of barley *Mildew resistance locus o, Mlo*) simultaneously deleted *TaMLO‐B1* and altered the promoter of the neighboring *TaTMT3B* gene, encoding a tonoplast monosaccharide transporter, resulting in both strong defense and reduced growth penalties (Li et al., 2022). Similarly, CRISPR mutagenesis of a rice susceptibility gene (*Resistance to Blast 1*, *OsRBL1*) leads to strong defense against fungus and bacteria but stunted growth with a 12‐nt deletion in its second exon reducing expression and balancing disease resistance with growth (Sha et al., 2023). These examples offer valuable insights into gene regulation and support the future design of plant traits.

Combining CREs with core promoter elements can be used to create synthetic promoters. Following numerous studies, defining a range of pathogen‐inducible CREs culminated in elegant work, building synthetic promoters consisting of tetramers of element types. They were found to mediate different strengths, speed of the response as well as distinct background levels in the interaction assays with a range of compatible, incompatible, and non‐host challenges and wounding responses. Next to demonstrating portability across species, the comprehensive testing also reported the impact of number, order, and element spacing and, with that, provided inspiration for the field (Rushton et al., 2002). The function of a common core sequence of CREs was investigated in tandem copies with different spacing and found to be conditional driving expression inducible by hormonal stimuli such as abscisic acid or salicylate (Mehrotra & Mehrotra, 2010). Cai and coworkers successfully predicted rational design and engineering of minimal synthetic promoters for constitutive expression by stacking CREs with a TATA box sequence and a minimal promoter core, including the transcription start site. Utilization of this method yielded a panel of synthetic promoters with varying strength (Cai et al., 2020). Beyond the well‐defined CREs, additional elements are increasingly recognized within the transcriptional units, which may modulate targeted expression (Boxes 1 and 2).

Box 1 **Main Points**

- Organ, tissue, and cell‐type specific expression regulatory elements (REs) are critical for current precision engineering.
- Advanced technologies in single cell analytics boost promoter identification at unprecedented resolution.
- Global identification of genomic regions harboring potential REs, integrated with *in vitro* mining of transcription factor binding sites are accelerants of the field.
- Combinatorial sets of high‐quality annotated genomic and transcriptomic data: an Eldorado for machine learning and artificial intelligence to discover new functional units of REs and transcription factors (TFs)?
- Element validation, design, building, and delivery of multi‐part constructs have progressed but remain a critical bottleneck as we begin to understand the profound impact of genome engineering and need to overcome transformation limitations.

Box 2 **Open questions**

- Can open‐source transformation SOPs/transformation facilities be adapted for elite lines, heterozygous, and self‐incompatible species of economic relevance?^1^
- Transformation technology: Can we fast‐track, standardize transient expression to validate parts and guide design principles for stable lines? Beyond genotype‐specific transformation technologies, portability and species‐agnostic tools are in need.
- Better prediction of engineered crop performance in downstream processing: many models cannot cope as engineered crops fall outside modeled parameters.
- Synthetic REs/TFs (i.e., nature‐inspired, elements insulated from the chassis' biological context, *de novo* evolved), a new frontier for comprehensive solutions and rational design of build‐test‐learn cycles?
- Scaling up and down: throughput and miniaturization. How to screen the massive output at the intersection of automated molecular biology and robotic analytics? Tissue and single cell‐type opportunities are recognized, but how to generically harness them?

^1^Note: From the breeding perspective, Saccharum and sorghum are self‐compatible, while the outcrossing, monoecious Miscanthus, and switchgrass are self‐incompatible as is the diecious poplar.

From the perspective of synthetic gene regulation, both endogenous promoters (for example the *SlWUS* promoter) and characterized synthetically assembled sequences (such as the hormonal responsive part above) can be considered as a single‐input YES gate, the efficacy of which is predominantly dependent on to what extent the single‐input matches the user's need. However, endogenous promoters and previously characterized CREs provide very limited specificity and strength. An excellent example is the *CSLF6* gene expression trade‐off introduced above. It is a formidable task to find a particular set of CREs to program high‐level expression with stringent spatiotemporal restrictions in the vegetative tissue type of a crop and before the harvest time. Nevertheless, these limitations can be potentially overcome by emerging synthetic circuit strategies (see below) that combine endogenous promoters and characterized CREs in a multiple‐input logic gate/circuit.

The characterization challenges and design considerations discussed above also apply to Polymerase III (Pol III) promoters, which are commonly used to express guide RNAs (gRNAs) in CRISPR research. Similar to Pol II promoters, Pol III promoters in published studies vary in length and often exceed 300–500 bp (Fauser et al., 2014; Ma et al., 2015; Xing et al., 2014), despite the fact that the native small nuclear RNAs (snRNAs) or CRISPR gRNAs they regulate are only ~100 bp long. The CREs necessary for Pol III promoter function are well‐characterized and include the upstream sequence element (USE, around −70/−60) and the TATA box (around−30) in dicots (Marshallsay et al., 1990; Vankan & Filipowicz, 1988). In monocots, one or more copies of the monocot‐specific promoter element (MSP) are also present upstream of the USE (Connelly et al., 1994). The close proximity of these CREs to the transcription start site suggests that long Pol III promoters are unnecessary for function.

Indeed, several studies have showed that U6 and U3 promoters can be shorten to 160–170 bp in monocots (Hao et al., 2020; Nagy et al., 2025) or 70–100 bp in dicots (Deguchi et al., 2024; Ma et al., 2015; Nekrasov et al., 2013) without affecting their activity. Hao and coworkers created a set of short (170–223 bp) Pol III promoters by artificially fusing 2–3 MSPs to minimal rice U3 and U6 promoters, which contained the TATA box, USE, and 1–2 copies of native MSPs, and showed improved cloning efficiency in multiplex construct assembly with editing rates comparable to those of native, longer promoters (Hao et al., 2020). Recently, Deguchi and coworkers experimentally verified a collection of 23 short (70 bp) Pol III promoters in stably transformed *N. benthamiana* and *Populus tremula* × *alba*. They demonstrated that sequences outside the conserved USE and TATA box can be freely mutated without affecting function, paving the way for new‐to‐nature Pol III promoter design (Deguchi et al., 2024). This concept is also applicable to monocot Pol III promoter engineering. Nagy and coworkers reported 37 computationally designed novel 500 bp Pol III promoters, with a 73% success rates when tested in maize protoplasts at full or shortened (280–300 bp) lengths (Nagy et al., 2025). The benefits of short, non‐redundant promoters in CRISPR multiplex editing are numerous, including simple and cost‐effective cloning via DNA synthesis of multi‐gRNA cassettes, reduced genetic load, and alleviation of potential silencing due to repetitive promoter sequences (Deguchi et al., 2024; Hao et al., 2020; Ma et al., 2015).

### Untranslated regions, upstream open reading frames, and terminators

Insertion of sequences upstream or downstream of coding sequences proved to effectively fine‐tune expression for more precise and improved bioengineering. Insertion of introns in the 5'UTR has shown to enhance expression in some cases, albeit with examples limited to monocotyledon species. This includes the maize alcohol dehydrogenase‐1 (*ZmAdh1*) intron in maize suspension cells and rice seedlings (Callis et al., 1987; Luehrsen & Walbot, 1991; Morita et al., 2012) and the sucrose synthase (*ZmSh1*) intron 1 additionally in *Panicum* and *Pennisetum* grass species (Vasil et al., 1989). From the perspective of cargo size, a 90% reduction to 150 bp was achieved and sufficient for full expression stimulation (Clancy & Hannah, 2002).

Another class of elements with regulatory role present, where investigated, on at least 30% of protein‐coding mRNAs, are upstream open reading frames (uORFs). Those carry additional putative AUG‐initiated translation starts. Recent technological advances (e.g., Ribo‐seq) have fueled genome‐wide discovery and growing interest in their distinct regulatory roles. As this topic is the subject of a recent comprehensive review, we would like the reader to refer to Wu and coworkers (Wu et al., 2024) for the complex cellular and molecular context. In brief, translation of a uORF may lead to interference with ribosome functionality before reaching the main ORF (mORF) or through mechanisms not yet fully understood in plants. As such uORFs may impact metabolic pathways, plant physiology and development through suppression, although examples of trait improvement were also reported (Wu et al., 2024 and references therein). As many of these uORF‐containing gene families are conserved across species, including the bioenergy feedstock poplar (Takahashi et al., 2020), they are excellent targets for genetic engineering. CRISPR knockout of uORFs has been used to boost mORF translation without affecting gene expression (Si et al., 2020), thereby diversifying strategies for manipulating agronomic traits. AUG sites in 5′‐leader sequences can support prediction of uORFs, where available (as described in Si et al., 2020), and enable their reporter‐based testing in protoplasts. Yet, their systematic targeting on global scale remains largely underexplored, plausibly due to an underrepresentation of cognate AUG sites, despite context of the flanking Kozak consensus. Indeed, in the wake of advances in Ribo‐seq technology for mapping of uORFs, non‐AUG, or non‐canonical translation initiation sites were identified at higher frequency than previously believed and may now represent opportunities for modulation of target translation.

Terminators and 3'UTR sequences also modulate gene expression, for example, the tobacco extension 3'UTR reduces gene expression (Rosenthal, Diamos & Mason, 2018), whereas the cowpea mosaic virus (CPMV) RNA‐2 3'UTR enhances it (see above in vector section, pEAQ‐*HT*). Characterized earlier in *N. benthamiana*, chimeric, synthetic 3′ flanking regions synergistically yielded a >150 enhancement of reporter gene expression over the nopaline synthase terminator. This effect is thought to result from reduced transcript read‐through and improved recombinant protein production (Diamos & Mason, 2018).

### Synthetic circuits

Given the knowledge gaps and slow pace of experimental validations in promoter characterization discussed above, strategies for developing synthetic promoters can offer alternative avenues. For a comprehensive review of inducible switches and gene circuit‐based plant programming, please see (A. Khan & Lister, 2025; Andres et al., 2019). Synthetic circuits can potentially expand tissue‐, cell‐, or condition‐specific expression patterns by creating new combinations, such as overlapping or exclusive expression (Brophy et al., 2022; Khan et al., 2024; Lloyd et al., 2022). Furthermore, even if a native element‐derived promoter achieves the desired expression pattern, such as pathogen‐sensitive activation (Xu *et al*., 2017; Liu *et al*., 2019), its transcriptional strength and spatiotemporal expression may benefit from fine tuning, or adaptation to desired responses. The synthetic TF mechanism is based on the classical operon model, where a DNA‐binding domain (DBD) is recruited to its DNA‐target. Coupling with a regulatory domain permits either activation or repression of the target gene. Schaumberg and coworkers developed synthetic TFs and performed quantitative assessments of their performance in Arabidopsis and sorghum protoplasts (Schaumberg et al., 2016). When multiple specific DBD‐DNA pairs are used—akin to bacterial repressor‐operator systems—synthetic TFs can regulate different genes simultaneously without cross‐interference. Comprehensive analyses of DBDs and their matching DNA sequences, allowed building circuits for tissue‐specific expression in Arabidopsis (Belcher *et al*., 2020). Microbial repressor‐operator pairs were assembled to create logic gates, achieving Boolean logic regulation in tobacco and Arabidopsis (Brophy et al., 2022). Transcriptional repression can be accomplished through DBD‐repressors or via CRISPR interference (CRISPRi), where a deactivated dCas9‐sgRNA binds to its target DNA without cleavage, guiding a fused repressor domain for gene silencing. This approach facilitated construction of logic gates demonstrated in mono‐ and dicot protoplasts (Khan et al., 2024). Beyond transcriptional regulation, so‐called ‘memory switches’ employ recombinases to delete a terminator sequence between the promoter and coding sequence of the reporter or turn off expression by cutting a part of the read‐out (Lloyd et al., 2022). A limitation of these ‘memory switches’, inherent to the recombination mechanism permanently changing sequences of the synthetic module has been approached through reversing the recombination (Bernabé‐Orts et al., 2020). Potential concerns of the cost of genetic stability at the target locus will need to be investigated in the future.

## PERSPECTIVES

### Field performance of engineered lines with gene‐specific promoters

Characteristics of modified traits in knockout, RNAi suppression, or overexpression lines, driven by ectopic, or unregulated promoters have been broadly tested (i.e., transferred) across species a spectrum of experimental conditions. Yet, few reports combine the assessment of bioenergy crops engineered with cell‐ or tissue‐specific promoters under variable abiotic and biotic plant environment, a.k.a. performance in field trials.

Coproduction of aromatic compounds (4‐hydroxybenzoic acid, 4HBA) was engineered in sorghum by driving a feedback insensitive variant of the committed step into the shikimate pathway through the maize *cellulose synthase* promoter, which is dominantly expressed in the secondary cell wall. Even though under field conditions two lines resulted in up to a 40‐fold increase in 4HBA over wild‐type plants, a significant yield penalty was found when compared to greenhouse‐grown plants (Lin et al., 2022). A concomitant reduction of up to 15% in biomass could indicate unexpected effects in the tissues dominated by secondary cell wall formation, which are the main determinants of plant weight. In switchgrass, modified for reduced recalcitrance of the cell wall to deconstruction, the stem‐specific promoter from the sugarcane *O‐methyltransferase* was used to express a bacterial enzyme, impacting lignin biosynthesis, while co‐producing an aromatic shikimate pathway derived metabolite (protocatechuate). Despite variation across lines, the biomass was found increased, matching findings of an earlier reported enzyme constitutively expressed in sorghum (Tian et al., 2022). In the engineered switchgrass lines, the gains in saccharification (sugar release) of 5% in average against the control in field samples were diminished in comparison to the earlier greenhouse study (18–24%). The earlier reported reduction in lignin content was also not supported by the field‐grown plants, with contributing experimental factors remaining speculative (Eudes et al., 2023). In poplar, a central enzyme of the phenylpropanoid pathway towards monolignols (4‐coumarate CoA ligase, 4CL) was suppressed by an antisense construct, controlled by the xylem specific *4CL1* promoter from aspen, and evaluated in field trials. In a range of lines differentially impacted for 4CL transcript accumulation, the correlated lignin content was found reduced, connected with phenotypical anomalies, yet without the expected improved saccharification (Voelker et al., 2010). Subsequently, the same target *4CL1* was suppressed using vessel and fiber cell‐specific promoters (from poplar *xylem cysteine protease 1* (*XCP1) and wood‐associated NAC TF 1B* (*WND1B*) genes, respectively). While both resulted in reduced lignin deposition, the vessel‐specific modification showed already in the greenhouse deformed xylem vessels and reduced sap flow. In the field, this translated to reduced height, biomass, and survival rate, with in contrast little to no penalty for the fiber‐specific modification (Cao et al., 2020). Direct comparison of greenhouse against field‐grown lines of transgenic poplar with low‐lignin genotypes demonstrated an environmental plasticity, that is, an increased total lignin content at all sites tested and plausibly compensating environmental stress (Stout et al., 2014). This environmental response held up as general phenomenon without regard for either constitutive or gene‐specific promoter used to generate lignin engineered poplar lines (reviewed in (De Meester et al., 2022)). In sugarcane, studies are focusing on plant resistance to biotic factors, with emphasis on deployed lines under control of a limited set of ectopic, non‐controlled promoters (summarized in Budeguer et al., 2021), while the literature for miscanthus indicates that research is directly advancing to gene editing without significant consideration of field trials of conventionally edited plants (Trieu et al., 2022).

These studies show that the power of greenhouse results for performance prediction in the field is limited. Hence, field validation will for now remain a decision gate in the advancement of the technology readiness level of bioenergy crops. Secondly, a multitude of factors contributes to phenotypic and performance variability under non‐controlled conditions. The rational selection, however, of the key functional parts, promoters controlling the engineered transcriptional units on cellular, or conditional level, will continue as first design element to guide downstream adaptation and fine tuning of the overall trait to match the specific technical requirements.

### Technical advances promise efficient circuit engineering; genetic part development

The performance of a synthetic circuit depends on the quality of its input, output, and regulatory components. To improve input accuracy and signal‐to‐noise ratio, classical enhancer trap and reporter analyses have been combined with advanced single cell sequencing and DNA library synthesis techniques, resulting in collections of promoters and CREs (Jores et al., 2020; Yaschenko et al., 2022). Similar high‐throughput methods have been used to identify other DNA elements, such as core promoters and terminators (Gorjifard et al., 2024; Jores et al., 2021), as well as regulatory protein domains from plants and other organisms (Morffy et al., 2024; Zhou et al., 2023; Hummel et al., 2023; Markel et al., 2024). The application of AI to gene regulation and engineering in crops is in its infancy, but has already generated promising results, with one example identifying a new order of CREs (Jevtic et al., 2024). On the near horizon, promising AI‐driven advances include redesign of Cas enzymes for improved activity or reduced size, improved sgRNA design, and integrated multiomics analysis to better understand engineering outcomes and iterate circuit design. This ongoing exploration of DNA and protein components expands the toolkit available to the plant synthetic biology community, enabling the design of more suitable inputs, efficient circuit devices, and functional outputs.

## FUNDING INFORMATION

This material is based upon work supported in part by the Great Lakes Bioenergy Research Center (GLBRC; FB, PC, AI, BRH), U.S. Department of Energy, Office of Science, Biological and Environmental Research Program under Award Number DE‐SC0018409; BRH AgBioResearch (MICL02454); endowment from James K. Billman, Jr. T. AI is supported by the National Institute of General Medical Sciences of the National Institutes of Health under Award Number T32 GM110523. This material is based upon work supported by the Center for Bioenergy Innovation (CJT; CBI), U.S. Department of Energy, Office of Science, Biological and Environmental Research Program under Award Number ERKP886. This work was funded by the DOE Center for Advanced Bioenergy and Bioproducts Innovation (CABBI, KS), U.S. Department of Energy, Office of Science, Biological and Environmental Research Program under Award Number DE‐SC0018420. This material is based upon work supported by the Joint BioEnergy Institute (JBEI; JM), U.S. Department of Energy, Office of Science, Biological and Environmental Research Program under Award Number DE‐AC02‐05CH11231 with Lawrence Berkeley National Laboratory. Any opinions, findings, and conclusions or recommendations expressed in this publication are those of the author(s) and do not necessarily reflect the views of the U.S. Department of Energy.

## CONFLICT OF INTEREST STATEMENT

The authors declare no conflict of interest.

## Data Availability

The data referenced in this paper are available from the comprehensive citations.
